# Quarterly Retrospect

**Published:** 1861-07

**Authors:** 


### *Quarterly Retrospect.*

*July*, 1861.

THE taking of the CENSUS in the course of the past quarter claims from us the primary, although but a brief, notice, in our Retrospect. The results, so far as at present ascertained, testify to the prosperity of the country. A parliamentary paper published on the 7th June, shows an actual increase of population to the extent of 2,169,576 between 1851 and 1861—an increase greater than in any previous decennial period; but the rate of increase, owing to active emigration, had somewhat diminished.

The recent publication of the *First Detailed Annual Report of the Registrar-General for Scotland* is worthy of especial note. As might be imagined, the report abounds with interesting facts and conclusions relative to the southern extremity of the kingdom, excellently set forth by Dr. Stark, and to several of these we propose to direct the attention of our readers, recommending them, however, not to rest content with the meagre illustrations to which of necessity we must confine ourselves.

The report is based upon the returns of births, deaths, and marriages for 1855. Touching solely upon matters in which Scotland presents some peculiarity as compared with other countries, or in which more light is thrown upon questions of vital statistics, and following the order of the report, we are arrested first by the facts relating to illegitimacy. The proportion of illegitimate births proves to be greater in Scotland than in England. This singular and unexpected result was first made known through the Quarterly Reports, and it has excited no small interest. It is curious also that in the distribution of illegitimate births in town and country districts Scotland shows a marked peculiarity.

"In almost all the Continental States," writes Dr. Stark, "whatever be the proportion of illegitimate Births among the rural populations, it is especially in the Towns that Illegitimacy acquires enormous dimensions. Thus in France, though the strictly rural population on a ten years' average only exhibited 4 per cent. of illegitimate Births, the 363 Chief Towns of France yielded 20·8 per cent. of the Births as illegitimate, while in Paris 32·8 per cent. of the Births were illegitimate. Take another instance. In Sweden, from 1850 to 1855, in the total population, 9·3 per cent. of the Births were illegitimate; but in Stockholm, during the same period, 43·3 per cent. of the Births were illegitimate. In Scotland, however, a somewhat opposite state of matters seems to prevail, inasmuch as in the Rural Districts on the Mainland the proportion of illegitimate Births is much higher than in the Town Districts. Thus, in the Town Districts, 7·1 per cent. of the Births were illegitimate; but in the Mainland Districts, the proportion of illegitimate children was 8·6 per cent. of the Births. The Insular Districts, however, proved a remarkable exception to this, and held what might almost be called the normal relation to the Towns, inasmuch as in them only 4·3 per cent. of the Births were illegitimate. This striking fact, then, of the inversion of the usual proportion of illegitimate Births in the Town and Country Districts, might induce the suspicion that Illegitimacy in Scotland and Illegitimacy on the Continent were to a great extent different things."

Again, in Scotland, the illegitimate births are almost solely confined to the labouring classes, and "the mothers of children chiefly consist of women employed about a farm or in agricultural labours, of factory girls, of domestic servants, and of persons engaged in needlework." Moreover, it is a remarkable, and as yet inexplicable circumstance that—

"Among every one of the above-named classes the tendency to illegitimacy is twice as great in the counties included in the North-Eastern and Southern Divisions of Scotland as among the corresponding classes of the great manufacturing and mining Counties which constitute the South-Western Division."

Illegitimacy, it is to be observed, curiously enough, is scarcely known among the inhabitants of the fishing villages.

The relationship observed between the distribution of illegitimacy over the surface of the kingdom and that of education, leads to one or two conclusions of remarkable interest. The returns showing the proportion of parties who signed the marriage register, make known, Dr. Stark states, the "apparently anomalous fact,—

"That the Counties which show the highest proportion of illegitimate Births are the very Counties which are in the highest condition as to education, in so far as such a conclusion can be drawn from the marriage register. And, on the other hand, the Counties which produce the fewest illegitimate births are those where education is, speaking in a general way, at the lowest ebb. . . . . . . . This striking fact then proves that the large proportion of illegitimacy in these highly educated Counties is not a sin resulting from ignorance and debasement; for any one who is acquainted with these Counties knows how intelligent are the natives, and that as moral beings, they are if anything of a somewhat higher cast than the generality of the inhabitants of those Counties where illegitimacy is at a much lower ebb. A minute examination of the marriage returns would probably prove this to be the case, and show that the true explanation was, that while the Counties in which illegitimacy was at a low ebb abounded in improvident marriages, the superior educational acquirements, and consequent more thoughtful habits engendered thereby, prevented these improvident marriages in the Counties where illegitimacy was high, but that, unfortunately, the moral training had not been carried so far as to enable them to master their natural passions."

This is a striking illustration, derived from statistics, that much of the education in vogue is seriously deficient in the elements of a sound moral culture.

Now, illegitimacy being most common among the rural population of Scotland, it becomes an important question whether "any circumstances exist among the agricultural population of those Counties where the illegitimate Births are most numerous, which act as a bar or check to Marriage to a greater extent than in those Counties where illegitimacy is less prevalent among the rural population?" The answer to this question is most curious, and affords a singularly interesting insight into the general circumstances of agricultural life in Scotland.

"It is a well-known fact," writes Dr. Stark, "that the smaller the size of the farm, the more it is worked with the assistance of unmarried young men and women, who are fed in the house, and either sleep in the farm-house, or offices, or, if the farm be larger, in bothies set apart for this purpose. In fact, it is only on the large farms that married men are employed; so that, in many districts, as soon as a young man marries he loses his situation as ploughman, and is forced to become a common labourer, dependent on his daily work for his daily bread. By a Return published in the Miscellaneous Statistics of the United Kingdom in 1857, we learn the number of farms in the various Counties of Scotland rated above £20, and the extent of land in each under a rotation of crops; and it is extremely instructive to see how closely, in many cases, the smallness of the average size of the farms in a County corresponds with the proportion of the illegitimate Births—the smaller the average size of the farms, the greater being the number of the illegitimate Births. Thus, in Banff, the farms paying £20 and upwards of annual rental only average 64 acres each; and it may be argued that, as a natural consequence, there can be few situations in such farms for married men, and therefore the illegitimate Births amounted to 13·6 per cent. of the total Births. In Aberdeen, the average size of the farms which pay £20 and upwards of rent yearly is only 66 acres, and hence we have 13·1 per cent. of the Births illegitimate. In Dumfries, the average size of the farms paying £20 and upwards is 87 acres only, and the proportion of illegitimate is 13·5 per cent. of the Births. In Kirkcudbright, the average size of the farms is 88 acres, and 12·0 per cent. of the Births are illegitimate. Take, on the other hand, a few of the Counties with larger farms, and on which a greater proportion of married men are employed, and compare the result. In the County of Edinburgh, the average size of the farms paying £20 and upwards is 114 acres, and among the rural population only 6·8 per cent. of Births are illegitimate. In Fife, the average size of the farms paying £20 and upwards is 110 acres, and only 6·6 per cent. of the Births are illegitimate. In Haddington, the average size of the farms paying £20 and upwards is 219 acres, and only 8·1 per cent. of the Births are illegitimate. Now, although these few startling facts convey some general idea of the apparent connexion between the size of the farms and the proportion of Illegitimacy, they fail to supply the fact which is chiefly wanted, and that is, the relative number of small farms—farms which are cultivated by the tenants themselves, or with the aid of one or two assistants. Any one can at once see from the above statement, that the state of matters on the large farms, on which alone the farm-servants are lodged in bothies, can have comparatively little influence on the proportion of Illegitimacy occurring in a County; and that it is not the large farms (which are comparatively few in number), and where the labourers lodge in bothies, but the small farms, which are laboured by the tenants themselves, or with the aid of one or two male or female assistants, who sleep in the house or offices, and are treated in all respects as one of themselves, which furnish the great proportion of illegitimate Births in the Rural Districts."

Other facts and deductions of interest are recorded in respect of illegitimacy, but we shall simply note in addition the absolute preponderance of females among the illegitimate Births in town districts. This fact, Dr. Stark points out, "affords the strongest possible confirmation of the truth of M. Giron de Buzareigne's conclusion, as to the weakening of the physical strength in the parents being the cause why the normal excess of male Births (viz., 105 males to 100 females) is not attained in towns."

The general education of the people of Scotland would appear to be in advance of that of England. In 1855, 29·5 per cent. of the Englishmen who married during that year, and 41·2 per cent. of Englishwomen, signed the marriage register with a mark. In Scotland, the same year, there was only one County (Ross and Cromarty) in which the number of men who signed the register with a mark was so high as the general average of England; and only three Counties, Shetland, Ross and Cromarty, and Inverness, where the proportion of women who used a mark equalled the common English mean. In not a single English county did the average of those who signed the register with a mark fall so low as 10 per cent., while in no fewer than twenty-two of the Scotch counties the proportion was even below that low average; and in two counties, Kinross and Selkirk, "every man who married was able to write his name in the register."

Consumption is the most fatal malady in Scotland, cutting off thrice as many individuals as Typhus Fever, which is the most deadly of all the epidemic diseases to which Scotland is subject. The mortality returns furnish most interesting information on the immunity of the Western Isles and the County of Argyll from the former scourge. Not dwelling, however, upon the detailed report on Deaths, we pass on to Dr. Stark's observations on the influence of temperature on mortality. His remarks on this subject are of considerable interest, although we cannot help suspecting that there is some confusion in his estimation of the effects of changes of temperature upon "epidemics" and upon "mortality." The two terms are by no means convertible, as Dr. Stark would in one or two instances seem to imply. Hence it is not improbable that a fallacy lurks under his conclusions.

"That the mortality, or number of Deaths, is greatly influenced by the kind of weather which prevails," he writes, "has been long known, but till recently this subject has not received half the attention which it deserves; and yet the influence of weather, but more especially temperature and amount of moisture in the air, both in the production of diseases and in increasing their fatality, is far more striking and far more powerful than any other known cause. One fallacy, however, which runs through almost all inquiries into this subject, has been the idea that atmospheric agencies must have the same action in all parts of the world, irrespective of climate, locality, and race. This, however, is by no means the case. That which observation may prove to be the law of the influence of weather on the mortality in Britain, will not apply to countries situated under different latitudes, or even in the same latitude, if the locality be greatly different. Each country, in fact, must endeavour to find out for itself the influence of weather on its mortality, as the laws which regulate it are modified by so many different circumstances from what they are in other countries. As a general rule, however, it will be found that countries which are adjoining, and whose atmospheric phenomena are, as a whole, regulated by the same laws, will conform more than those whose atmospheric phenomena are directed by different agencies.

"It is from neglecting to make this discrimination that we find it constantly asserted by writers, that 'epidemics seem to be generally combined with summer or hot weather;' and hence, without examining the facts for themselves, sanitary inquirers in this country repeat the same stereotyed phrase, and attribute every epidemic to the existence of fetid exhalations from drains, cesspools, &c. Were this the case in this country, it would be found that epidemics would chiefly rage when the heat was greatest, when alone these putrid emanations are rife; but the fact stare us in the face, that, excepting one single class of diseases—viz., Bowel Complaints—the prevalence and fatality of almost all other epidemics in this country abates, and is at a minimum, during our warmest months; and the still stronger, because direct fact, is now clearly proved, that the prevalence and fatality of all the ordinary epidemics in this country *increases with the increase of cold*—increases as the supposed putrid exhalations become less virulent, or are quite arrested."

Dr. Stark then examines the progression of temperature and mortality in Scotland in 1855, and thus proceeds:—

"Now, supposing it be taken for granted that the year 1855 manifested the usual influence in temperature on the mortality of the inhabitants of Scotland —and it quite agrees with all we know on the subject—then we have not to dread an increased mortality with a rise of temperature as high as we usually have it, but we have to look in every case for a reduction in the number of Deaths. The fact is, the monthly mean temperature in Scotland very rarely rises above 60° even during our warmest months, July and August; and it is only when the temperature keeps *at or above* 60° for any considerable period, that any tendency is ever observed to an increased mortality. When the temperature, however, keeps at or above 60° during July and August, a slight increase of the Deaths is observed, solely dependent on Bowel Complaints, viz., Diarrhœa, Dysentery, and Autumnal Cholera; but in no case, so far as yet observed, dependent on other epidemics. These Bowel Complaints are the epidemics in Scotland which increase with increase of temperature; almost all the other epidemics seem subject, to a very great extent, to the same laws which rule the ordinary Deaths, viz., they increase in number as the temperature falls below 50° Fahrenheit. Unfortunately, from the want of a sufficient statistical staff, this important fact cannot be shown for 1855; but the fact has been pointed out for other years, so that there can be no doubt on the subject.

"If such be the case with Scotland, how, it may be asked, has the statement originated that epidemics, and of course increased mortality, are generally associated with summer or hot weather? If we look to the influence of season on the population of those countries which are situated in warmer latitudes, and which enjoy a continental climate, the explanation is at once apparent. With them, the cool months, as might be expected, are the healthy ones, while the warmer months are the unhealthy ones. And even the observations made in these favoured temperate lands suggest the cause of this; for even here we find that the moment the mean monthly temperature rises above 60°, diseases are induced by the heat which prove fatal to the population nearly in the exact ratio of the rise of mean monthly temperature above 60°. The accompanying Table, showing the monthly Deaths in the City of New York and the State of Massachussetts, in the United States, for a series of years, together with the mean monthly temperature of the same years as taken at New York, will serve as a good example of the baneful effects of a rise of mean temperature above 60° on the health of the population. Here it will be observed, that while the mean temperature rises from the freezing point the Deaths diminish, as in Scotland, till the mean monthly temperature reaches 60° Fahr.; but from the moment that the mean temperature rises above that point, the number of Deaths begins to increase, and during the warmest months attains a maximum nearly double that of the cooler months, and continues high so long as the mean temperature is above 60°. As the diseases induced by a high temperature, however, take some time to run their course, the first month when the mean temperature rises above 60° does not exhibit anything like the full amount of the destructive action of the heat; and from the same cause, the cooler month, which succeeds the hot ones, contains among its Deaths a considerable proportion of those whose diseases were induced by the hot weather. Hence at New York, June, though a hot month, exhibits but a slight increase of Deaths, while in July and August the number of Deaths is nearly doubled; and it is not till the cool month of November that they have subsided so far as to allow the minimum mortality for the year to be attained. With the setting in of winter, however, the Deaths again increase, as in this country,—attaining their winter maximum in March, as in several of the Continental States of Europe. In these warm regions, however, where the summer or autumnal mortality rises so very high, the winter maximum mortality never nearly approaches that of the hot season."

The question of founding a Benevolent Asylum for the Insane of the Middle Classes has, during the quarter, once more been revived. Fifteen years ago an attempt was made to establish such an institution, but it failed—probably in consequence of the commercial panic which occurred about that period. On the 19th of April last, a public meeting was held at the Freemasons' Tavern for the purpose of forming a Society which might carry out the object referred to. The Earl of Shaftesbury presided, and the meeting, which was numerously attended, was addressed by the Chairman, Lord Ebury, Mr. Watlington, M.P., Mr. S. Cave, M.P., Dr. Conolly, Mr. Tite, M.P., Mr. Solly, Dr. O'Connor, and others.

In reviewing the proceedings of the Select Committee on Lunatics which sat during the two last sessions of Parliament, we commented pretty fully upon the advantages to be derived from the establishment of an asylum such as is now proposed. The subject was entered into at length in the evidence given before the Committee by the Earl of Shaftesbury and other witnesses, and, although deprecating the manner in which needless and unjust aspersions of the proprietors of private lunatic asylums were intruded into the question (an error not even avoided in the meeting at the Freemasons' Tavern), we cordially approved of the object aimed at. It is not necessary that we should travel again over the ground we then traversed. Asylums of the class sought to be established are much required, and every effort to procure them will be certain to receive the warmest aid of the medical profession, especially of those members of it practising in lunacy, since they chiefly are acquainted with the evils arising from the want of an institution which would supply all the care of a well-regulated asylum at a lower cost than is practicable in a private asylum. There is a large class of persons, consisting of small tradesmen, governesses, clerks, literary men, half-pay officers, the poorer clergy, &c., who, under existing circumstances, in the event of mental alienation, are deprived, to a great extent, of all such assistance as would best befit their cases; for the county asylums are closed to them, as a rule, except as paupers, and private asylums are beyond their means. To supply, therefore, the wants of this class, asylums are wanted founded upon a substratum of benevolence. To attain this object is the aim of the Society happily formed by the Meeting held on the 19th April. In opening the meeting, the Earl of Shaftesbury observed that, "They proposed to begin with but a small demand. The number of patients for which it was proposed to make provision would be in the first instance but few. Perhaps, when they first appealed to the public on the subject fifteen years ago, they asked too much, and it was not intended to err in that direction on the present occasion. What they asked now was, sufficient to purchase a piece of ground with a suitable house upon it, and to adapt that house to the purpose of the asylum, commencing the experiment with only a small number of patients."

We trust that the fullest success will follow these efforts.

In 1856 some curious facts were made known, in a Parliamentary Paper, respecting the diet of the convicts at Fremantle, in Western Australia. It appeared that while our army before Sebastopol was being decimated by starvation, our convicts at Fremantle were being decimated by over-feeding. The unfortunate convict was literally over-gorged with food. After making allowance for *loss sustained in cooking*, the solid aliment he received exceeded by one pound the full diet estimated by competent authorities to be required by a labouring man in England. As a consequence, unless when he was placed under a curtailed regimen by way of punishment, the convict was constantly beset by

> "Dropsies and asthmas, and joint-racking rheums."

Now, this over-gorging was no accidental matter, neither did it arise from any misplaced kindness towards the convict. The authorities had found, in fact, that he was more readily manageable when fed to repletion. He became, indeed, exceedingly susceptible to any stint of food, and consequently the ordinary punishment of low diet proved a highly effectual curb to unruliness or obstinacy.

We are reminded of the *gluttony plague* (as it has been aptly termed) among the convicts at Fremantle, and of the reasons which led to their being over-fed, by the recent serious mutinous outbreaks among the convicts at Chatham. Popular opinion has sought an explanation of these disturbances in the theory that the convics there are pampered. If this be true, the contrast between the results at Fremantle and Chatham are not a little curious. But be this as it may, the convict riots at the latter place have, justly or unjustly, somewhat disturbed the happy confidence we were indulging, and have expressed from time to time in these pages, in the efficiency of our convict discipline, and its immediate and prospective results. The *Times* (April 18th) has the following suggestive remarks upon the subject:—

"Signs are abroad of an impending change in the popular view of the great 'Convict Question;' and, indeed, it would be hard to find any subject on which the ordinary reflux of opinion has been delayed for so long a time. The social history of this country has been called a history of reactions, and, though the description is exaggerated, it is not without a basis of truth. It is in our nature to obey one impulse after another, but the fact is that in this particular instance there have been but two currents of feeling, and the reaction has been long and steady, because the action which produced it was proportionately forcible. From the earliest times up to the reign of King-George IV. we treated not only our convicts, but even our prisoners often with great inhumanity, and always without the least consideration. It can hardly be said that the system was much mended either by the inquiry which Hogarth has commemorated, or by the noble efforts of the philanthropic Howard. Things remained almost as bad as they could be till the days of Wilberforce and Romilly. The gaols, indeed, grew somewhat better, but the gallows still offered the chief release from the gaol. In front of those black walls of Newgate men and women were strung up by the half dozen, and the visitors to that grimy old prison may listen to St. Sepulchre's chimes as Ranald MacEagh listened to the alarm rung out from Argyll's castle, and think of his remark to Dugald Dalgetty, that 'at the sound of that bell many a brave man had yielded up his soul.' In fact our Convict Question in those days had but two solutions—Botany Bay and the halter. There was a cruel simplicity about the system. Society cared a good deal for itself, nothing for its sinning members. The mischievous classes were put out of the way with little trouble, and even less compassion. Perhaps they were transported, perhaps they were strangled; but in either case they ceased to be troublesome at home. True, the crop of crime always ripened afresh, but the sickle was as ready as ever. Nobody concerned himself either about gaols or gaol deliveries, about reformation, classification, or superintendence. 'So,' as Mr. Dickens says in the latest number of his latest work, 'felons were not lodged and fed better than soldiers (to say nothing of paupers), and seldom set fire to their prisons with the excusable object of improving the flavour of their soup.'

"With the mitigation of the criminal law came the turn of the tide which has been flowing ever since. Capital punishment was gradually disused till it became the penalty of murder only, and not always of that. Still, we transported our criminals freely beyond seas until a fresh obstacle was encountered. Our colonies refused to receive these tainted cargoes, and we found ourselves compelled to keep the culprits at home. Then came Penitentiaries, Convict Prisons, and other establishments of the like character, followed by Reformatories, Model Gaols, and a variety of institutions for improving criminals and eradicating crime. As convicts could no longer be either hanged or transported, and as it would not do either to let them loose or keep them locked up for ever, the only alternative was to reform them, and we set about the work, not only for humanity's sake, but because there was nothing else for us to do. But when the current of public feeling had once set in this direction it naturally overflowed its bounds. So much was done for our prisoners that they became better off than honest men, and theft led to comfort by a shorter road than industry. The latest phase of the affair is a convict revolt, created apparently by a fine stimulative dietary, *apropos* of which an intelligent official writes to us much as Paterfamilias writes about Eton, and represents that criminals should not be crowded together in such large numbers, but be distributed, as it were, into snugger classes, and under more tutors or superintendents.

"However, there is a change impending at last, and a stronger sign of it could hardly be found than in the tone adopted by so known a philanthropist as Mr. Charles Dickens. It is curious enough to find the author of *Barnaby Rudge* penning such a sentence as we have quoted above, but the feeling is perfectly justifiable, and the only question is what to do next. Our readers have already learnt something of the conflict between the English and the Irish 'Convict Systems,' and have seen that the officials are firmly convinced not only of the humanity but of the 'success' of our recent courses. We believe, indeed, that up to a certain point their opinions are defensible. We have no doubt that many criminals have repented in our Penitentiaries, and been reformed in our Reformatories, but we are perfectly sure also that in many cases all our care has been thrown away, and we feel by no means easy about the numbers of convicts who are periodically turned loose upon society, to sink or swim. According to Sir Joshua Jebb's statement, about eight go right where one goes wrong, or, rather, only one out of nine can be shown to have relapsed. This is certainly not a result to be ashamed of, but there still remains the percentage of incurables, and it is with these that we are mainly concerned. In a few words, our belief is that a certain proportion of convicts is, and always will be, utterly irreclaimable by any measures we can adopt at home. We do not believe in any possible progress either of the 'English' or the 'Irish' system by which these men can be reformed. We think that they will continue to defy our efforts and produce an immense amount of evil, and we consider that in their interests as well as our own, they should be sent off to some of the districts of Australia where the settlers seem still to be not only willing, but anxious to receive them.

"Let the reader only look at the cases which are perpetually repeated in our Law Reports—at the proceedings, for instance, at the Middlesex sessions this very week. After a prisoner is found guilty, inquiry is made, as a matter of course, respecting his previous doings, and it frequently transpires that he has been tried and sentenced half-a-dozen or a dozen times before. No fewer than fifteen convictions were proved on Monday last against a young lad of eighteen, and we have seen cases in which that number was doubled or even trebled. Now how is it to be expected that such characters as these should yield to the discipline of Reformatories or Model Prisons? It need not be assumed in every case that there is some portentous degree of vice to be overcome. There is, perhaps, nothing of the sort, but there is something still harder to deal with—the spirit of an attractive profession. These inveterate offenders are experts in their craft. They are skilled labourers of the highest pretensions. A set of housebreaking implements produced before the Assistant-Judge on Tuesday were such models of artistic manufacture as to fill even the police with admiration. Depredation, in fact, has been refined into an art, and every art has its slaves. It is hopeless to expect any better things from these unprincipled proficients so long as they remain at home. They prey upon the community by irrepressible instinct until they are detected; when detected they take the chances of trial; when sentenced they accept their penal servitude, knowing that it will have an end; and when released, probably by a premature discharge, they return to their malpractices with greater zest than ever. These men constitute that ugly percentage of convicts with which nothing can be done—the true blackamoors of the system who can never be washed white. Here it is, and perhaps here only that we fail. We can do an infinite amount of good by prevention—witness Ragged Schools and 'Industrial' Institutions. We can do some good by cure where nature happens to be favourable and disorder has not gone too far—witness the reclaimed delinquents to whom Sir Joshua Jebb points with such justifiable pride; but there are cases in which we can do no good at all—witness the scores of offenders brought up for their fortieth or fiftieth offence, and our best course would be to ship off these incurables to a land where they might do some good, and where, at any rate, they could do no harm."

Among the criminal events of the quarter several murders are recorded, committed apparently by lunatics. The consideration of these cases, however, we postpone until after the trials of the murderers.

The following singular and novel illustration of the criminal propensities observed among the insane, is of considerable importance to the forensic physician, and we place it on record without curtailment, adopting the report contained in the *Daily Telegraph* of April 5.

"At the Birmingham Quarter Sessions on Wednesday (April 3), a young woman who gave the name of Eliza Davis, described as the wife of a respectable professional man in Birmingham, but whose real name did not transpire, was charged with having, on the 28th of February last, feloniously and by fraud taken away a child, the son of John Doon, with intent to defraud the said John Doon of the possession of the said child; and also with having on the same day feloniously taken away a young child, the son of John Brown, with intent to defraud the said John Brown of its possession.

"Mr. Manley Smith appeared for the prosecution, and Mr. O'Brien, instructed by Mr. Edward Powell, for the defence.

"In opening the case, which was of the most extraordinary character, Mr. Manley Smith said it was laid under the 9th Geo. IV. cap. 31, sec. 21, by which, if any person maliciously, by force, or fraud, deprived a parent of the possession of his child under ten years of age, he or she was made guilty of a felony, and liable to transportation. He then proceeded to detail the circumstances of the case, after which he called

"Eliza Macarthy, who said she was the wife of John Macarthy, shoemaker, Henrietta-street. On the 28th of February she saw the prisoner at the gates of the Snowhill Railway Station, when she was asked by her if she had seen a woman with a baby, and said she had not. The prisoner then asked if witness knew any one with two babies, and she said she did not. Prisoner accompanied her towards home, and met a woman at the corner of the entry, to whom she said something about two children, and that any one who would find them would do her a favour. That was about seven o'clock in the evening. The woman they had met said there were children in the street, and took her to Mr. Brown's door. The prisoner went in and asked if Mrs. Brown was newly confined. Mrs. Brown was in bed, and the child, which was a month old, was with her. Witness did not hear the whole of the conversation, but Mrs. Brown said she was afraid to let the child go, and to trust it with the prisoner. Brown then went out, and soon after came back with Mrs. Doon and her child. When they came, prisoner said she would rather take the two children; and Brown said, 'Let us be quick about it, and get it over, as it is getting late.' After prisoner went up-stairs she said she had been seduced by a gentleman at Liverpool, was pregnant by him, and had told him that she had had twins. Prisoner said further, that he wanted to see the children, and he would give her 1000*l.*, and take her home to her father and mother. She said she would not want to keep the children more than half an hour. When Brown had agreed to let the children go, witness carried Brown's child, Mrs. Doon carried her own child, and Brown went with them to Nock's dining-room. Prisoner went in first, and came out almost immediately, and said he was not down-stairs. She then went up-stairs, and soon returned smiling, when she said he was up-stairs, but was deep in conversation with another gentleman, and seemed frightened, and said, 'Jemima, are you going to have me taken? What are all these people about you?' She also said he had told her to walk down to the New-street station. Prisoner took the baby witness had been carrying, and they all went to the station, where witness and Brown went to one side, and the prisoner and Mrs. Doon on the other. Witness never saw the prisoner afterwards, as she made her escape with the children.

"Cross-examined: Prisoner was very anxious about the child, and cried when she thought Brown would not let her have it. She said one child would do at first, but when she saw the other one she said she must have them both.

"The husband of Mrs. Brown and Mrs. Doon were then called, and confirmed the facts above stated, connected with the application of the prisoner to be allowed to take the children, and her subsequent disappearance.

"Mr. Hawkins, carpenter, Tenant-street, said that on Friday, the 1st of March, about one o'clock in the morning, he heard some children cry, and looking out he saw the prisoner with two children. He asked her what she wanted, and she said she would tell her business if Mrs. Hawkins would go down to her. Mrs. Hawkins went down-stairs, and finding that both the prisoner and the children were very cold, made up a fire, made the prisoner some tea, and fed the children. The prisoner said she had been confined at the Birmingham Union, and that she had left with her two children because she could not bear to have them confined in a separate room. In a little time the prisoner made several excuses as reasons for going out without the children, and Mrs. Hawkins became suspicious that she wanted to leave them. Prisoner said that her parents were named Hill, and lived in the Hagley-road, and that she had been seduced by a young man, a pearl button maker. She had gone to the Union to be confined, so that her father did not know of her shame, and that she durst not take the children home at once lest he should beat her. She therefore asked to be allowed to leave her two children with Mrs. Hawkins until she had been home and explained the matter. Witness would not agree to this, but said that if the prisoner would stop till a reasonable hour next morning, Mrs. Hawkins should go with her home, carry one of the children, and see what her parents would do for her. The prisoner objected to this; and having had the children warmly wrapped up, she left the house of witness with them about half-past five o'clock that morning.

"Mrs. Dugmore, Harborne, said that about a quarter after seven o'clock on the morning of Friday, the 1st of March, the prisoner came to her house with two children, which she wanted witness to nurse for her. She said they were the twin sons of her sister, who had died a month before; that they had been with another nurse, but when she went to see them they were in such a neglected state that she at once took them away. Witness said at first that she could not take them, but at last agreed to take them for a day or two. On the Saturday night prisoner sent a cab for her, and met her at the Five Ways, when she said the children were her own; that she had been confined at the Union; that a lady was going to give her 50*l.* for one of the children; and that she was going to have the other nursed by a poor woman. Witness said that she could not keep the children under such circumstances, and the prisoner said she would send for them on Sunday or on Monday at the latest. On Saturday the parents of the children came to her house and took them away.

"Mr. O'Brien, for the defence, said there could be no difficulty in this case, as the jury saw before them, not a criminal, but a woman who was greatly to be pitied, as she was suffering under a mental derangement. She was the wife of a respectable professional man, had a comfortable home and an affectionate husband, but unfortunately was at times subject to such fits of delirium as that under which she had in this case taken away the two children. The very cunning which she had exhibited in every stage of the affair was only, as must be seen by every one of them, the cunning of madness. He then called

"Mr. Bull, who said he was a surgeon, living in Birmingham. He knew the prisoner, whose name was not Eliza Davis. She was the wife of a medical man in this town, had a good home, an affectionate husband, and three children living. Witness had attended her professionally in her confinements. Her youngest child was about two years old. On various occasions she had suffered severely from mania; and witness had attended her several times for affections of the brain, when her husband had called him in. On these occasions she exhibited indubitable symptoms of derangement. When about to be confined, she had on two or three occasions contrived to get every one out of the way and make her escape; she had twice been confined away from home in that way. From what he had heard said by the various witnesses, he should say, without the slightest doubt, that at the time she took away the children she was insane.

"Mr. William Moseley Richards said he was a surgeon, living in this town. He had known the prisoner for some time. She was the wife of a professional man in Birmingham, and had a comfortable home. Within the last two years she had repeatedly been in an insane state of mind, the symptoms of which were a partial incoherence, restless manner, strange language, in short, similar to those produced by delirium.

"In summing up, the Recorder said it had been clearly proved that the prisoner had brought herself within the meaning of the Act of Parliament, but there did not appear to be any doubt but that she was insane.

"The jury acquitted the prisoner on the ground of insanity."

The following is a summary of a remarkable case of suicide and attempted suicide which occurred in Paris at the close of last year, the judicial proceedings connected with which terminating in the present quarter. It illustrates a state of society and feeling almost peculiar to our neighbours.

Corporal Rouard had filled the office of librarian to his regiment, and his seven years' term of service having expired, he voluntarily determined to serve for another term, and received the re-enlistment of 1,000f. Finding himself in possession of a sum of money such as he never saw before, and never could hope to see again, he determined to "knock out life" with it as long as it lasted. The story of his saturnalia is told by the following summary of the report of the advocate-general to the Paris court-martial:—"On Dec. 2 last Rouard made the acquaintance of Denise Herbin, an artificial flower maker—a pretty girl, only seventeen years old—at the dancing rooms called the 'Grand Salon,' in the Faubourg Poissonnière. Rouard had a lodging in Paris, in which he installed Denise, and he gave up to her every moment that he could spare from his military duties. As long as his re-enlistment money lasted, which was only a few weeks, they led a joyous life, and troubled their heads little about the future. He had been guilty of deceiving her as to his position in life; for, instead of admitting that all the wealth he had in the world consisted of his re-enlistment money, he had represented himself as having some private income, and had promised to allow her permanently 50f. a month. This made it the more painful for him to break with Denise, which he felt it absolutely necessary to do. On the last day of his leave of absence, he began by asking her, "In what way do you love me?" She gave a true woman's answer: "I love you because I do love you." He afterwards repeatedly told her that she must leave him; but they at length agreed to die together. They made up their clothes into separate bundles, which they ticketed with the names of the relations whom they intended to have them, and then Rouard wrote three letters: one to his father, another to his sergeant-major, and a third to a sister of Denise, announcing their intentions. Denise asked Rouard what sort of death they should die? "We will die," he replied, "as we began. We were first intimate at the Cadran Bleu, there let us finish our career. I will plunge the poniard into your bosom, and I will then draw it out all bloody and stab myself." Rouard says that by drawing this bloody picture of death he hoped to frighten her and divert her from her purpose, but that she, far from being daunted by his words, flung herself into his arms in ecstasy, exclaiming, "I could never have hoped for such an honour." At six in the evening, Rouard accompanied Denise to the shop where she worked, that of Madame Vielle, artificial flower manufacturer, Rue St. Denis, and there she distributed her working tools among the shop girls, kissed them all round, took a solemn leave of them, and raising her hand in the way which French people do when they take an oath, said, "I swear before God, and by the ashes of my mother, that I am going to kill myself." The people in the shop, who had always looked upon Denise as rather flighty, did not for a moment suppose that there was anything serious in this oath. On leaving the flower shop, Rouard and Denise went to the Bal Robert, where they danced till nine o'clock, and then went to the Cadran Bleu; but finding that establishment closed, they proceeded to Michel's Restaurant, nearly opposite, and asked for a private room, pens, ink and paper, and supper. On entering the private room in the restaurant the poniard was placed on the table, and Rouard said to Denise, "The truth must be known after us; I should not like to be thought an assassin." Thereupon Denise wrote, "I die, struck by the hand of my lover; I have ordered him to do so because I wish to die with him;" and she signed her name. Rouard wrote underneath the following words: "I stab my mistress because she wishes it. I die with her because such is my will." After supper, and when the minute hand of the clock pointed to eight minutes to eleven, which was the exact hour they had fixed upon for the completion of the drama, Rouard said, "The hour has come." Denise then laid down upon a sofa, undid the upper part of her dress, and bared her bosom. Rouard took the poniard, advanced towards her, and said, "It is yet time,"—meaning to ask if she would change her mind. She replied, "Go on; and, above all things, don't make me suffer." He then placed one knee between her legs, pushed up with his left hand her left breast, and plunged the weapon into her body up to the hilt. She heaved one sigh, closed her eyes, and remained motionless. Rouard drew out the poniard, and, while warm and bloody, as he had said, inserted the point into his own bosom; he then placed the handle against the body of Denise, and folding his arms around her, pressed her against it with all his strength. It was deposed by witnesses (for Denise did not die on the spot) that she subsequently affirmed, when in the hospital, that, "feeling her lover tremble, she pressed herself against the handle of the poniard as hard as she could, in order to help to drive it home." After this Rouard fainted and fell off the sofa. When he came to himself, and sought to collect his ideas, he felt an acute pain in his breast, and found the poniard sticking in it. He drew it out, laid it on the table, and then sat down on the sofa where Denise was lying. It being now late, the waiter knocked at the door of the room, and Denise had strength enough to stretch out her hand from the sofa and draw the bolt. Both Rouard and Denise, severely wounded in the lungs, were removed to the Hospital Saint Louis, where Denise lingered for twenty days. Rouard sufficiently recovered to take his trial. A case of the above kind commonly results in a triumphant acquittal before a French jury. A court-martial, however, might be supposed to be made of "sterner stuff." In this case, however, the majority of the military tribunal showed itself susceptible of those feelings which, though admitted by no civilized code, generally give impunity to crimes of this nature in France. Rouard was acquitted by five votes against two. His mother, who was in court, loudly thanked the judges, and there was a general shout of applause.

We conclude our Retrospect with the judicial record of an instance of superstition and crime almost unparalleled in the present day. The particulars were communicated to the *Daily Telegraph* (April 3) by a correspondent at Gratz. He writes:

It seems that a few months ago, a peasant of the name of Lauder appeared before the district court of Leohen, in Styria, on the charge of having murdered a certain Frau Catherine, whom he believed to have been a witch. A second peasant, whose name is concealed under the initial R., was accused of having abetted the other by advising him, on the faith of a pack of cards, to commit the deed. Lauder is a young man of 27 years, the father of a family, and of respectable antecedents. R., an old beggar, aged 75, who appeared in rags before the court, looked, it is said, as much a sorcerer as the poor victim of his superstitious practices.

On being called upon to reply to the charge, Lauder spoke as follows:—

I ask your pardon, gentlemen of the court, for not being able to tell the story in a very connected manner. I always suffered from a bad memory, in consequence of my father having beaten me too much when a boy. When four years old, I was put to work, and on one occasion, as I have heard from my mother, he knocked me down, and so hurt me that I was laid up for a long time. After his death, my mother married again, but my step-father behaved still worse, and several times was very near being the death of me.

President of the Court.—I should wish you to come to the point, and tell us how you were the death of Catherine.

Lauder.—I killed her, because I had been ill for a long period. Gentlemen, I beg you will give me time. The fact is, I have always been ill. My wife, being very skilful in charms, she cooked different sorts of herbs for me, which made me better. Some time ago, when suffering from a swelling in the eye, a cobbler advised me to look for the bone of a dead animal in the fields, when the moon was on the wane. When I had found it, I was to take it up with my handkerchief, and cross it three times over my eye. I was lucky enough to meet with a bone, and, doing as I was ordered, my eye soon got well. I have a right, then, to believe in charms. But, on returning a shoe which I had mended for Catherine, the swelling came back. Directly I entered her room I felt a sharp pain in my leg, which ran up to my head, till I could hardly bear it any longer. I told my wife that, after all, there might be truth in the "planet book," which, on looking after my name, and comparing it with the position of the stars, clearly showed that I had been bewitched. My wife, who knew all about these matters, advised me to go to R. for advice. She is a clever woman, I must tell you. Once, when the goat was losing its milk, she boiled a pot full of it while the bells were ringing for prayer, and the goat soon got back its milk. I therefore entirely relied upon my wife's opinions, and went to the man R., the same who had correctly foretold the death of my father-in-law. On consulting the cards, R. was speedily convinced that Catherine was the witch who had buried a "nail of death" in front of my house. We went to the spot, and dug up all around without finding the nail. I then told R. that I did not want to know anything but the real truth. He looked again at his cards, and after some time gave it as his opinion that she had poured out water in the form of a cross, calling my name all the while with curses. He also counselled me to go to the old witch and beat her severely, when, perhaps, she might be prevailed upon to undo the spell. My wife objected, but on R. saying that this was the only means of recovery, I at last resolved to go and do as I was bid. Gentleman, when a worm defends its life, it is natural that I should not give up mine without a struggle.

On Catherine opening her door, I instantly called out to her, "You cursed witch! why have you charmed me, who never did you any harm?" She replied, "Nor have I ever done you any harm." Well, as R. was certain that she had committed the mischief, I caught her by the hair, and knocked her down. Catherine begged and prayed for mercy, saying that she would undo the spell if I would let her off. Upon which I asked, "Will you really let me off?" when she said, "I have never done you any harm." This put me in a great passion, as I cannot bear lies. Thinking that I should compel her to keep her promise, I beat her to death. I was in my senses all the time.

President.—Was any one else present?

Lauder.—Yes, there was a stranger. The man said that he had never believed in witchcraft until he saw a man in Austria (the Archduchy) who was wasting away from being bewitched. The man also acknowledged that I was in the same case, and regretted that he could not undo the charm.

President.—What did you do after killing her?

Lauder.—I went home with my wife, who had been waiting in front of the house. I did not think that I had quite killed her, but, to make sure, asked R., who had also been waiting outside. He could not tell till he had looked at his cards. "She will come round again, you may be sure," he said; "such a witch has a devil in her, and is not so easily killed." But as I had my doubts about the matter, I proposed to go to the priest and have a mass read for her soul; and though R. advised me not to tell any one, not even the priest, I didn't mind him, and went. When I had told him what had taken place, he said:—"You stupid man, how can you believe in witches? If you do that, masses are of no avail." I then asked what I should do, when the priest replied, "You had better go and look for the doctor." I did so; but, on going with him to Catherine's house, another doctor met us halfway, and said that she was already dead. I was rather unwell that day, but on feeling a little better towards the evening, I thought I might as well go to the police office, and give an account of it all. But on second thoughts, being pretty certain that the police would come to me, I stayed at home. And they did come very soon.

President.—And had you no notion that you were doing wrong by killing the woman?

Lauder.—I thought I might defend my life, when she was going to take it. If a robber were to attack me, there is no doubt I should be allowed to keep him off; and am I not to be allowed to save my life, when somebody is going to murder me? Besides, I did not mean to kill her, but merely intended to give her a good sound beating.

President.—Every person in the village says that she was a particularly good-natured woman. Did you not know this also?

Lauder.—My wife, who is very clever in all these things, assured me she was a witch. Somebody had told her on the Alps that Catherine knew how to change horsehair into otters.

Public Prosecutor.—On another occasion you called R. a cheat and a deceiver, because, immediately after the commission of the crime, he proposed a different method of attaining the same object.

Lauder.—That is true. He advised me to cut the top of three juniper bushes, to lay them crosswise under a large stone in front of my house, and pour over it some Midsummer-night's wine.

Public Prosecutor.—And do you still believe in such charms?

Lauder.—No, I do not. The fact is that that man R. has been making a living out of such practices for many a year. He told me also that if he did something in front of my house, and then repeated the Lord's Prayer three times backwards, I was certain of being a dead man within a year. Collier Fritz, they say, is also a man that can undo charms.

President.—Do you think your deed was a good and right one?

Lauder.—Had I known that it was wrong, I should certainly never have committed it.

The President then proceeded to the examination of the old man R., the accomplice, who, among many other things of minor importance, deposed as follows:—

R.—The cards told me that a nail of death had been placed before his door by a witch. I instantly suspected Catherine, having heard her threaten the priest's cook with witchcraft on another occasion. Soon after the priest's cows fell very short in their milk.

President.—Are these the cards you used on the occasion?

R.—Yes, they are.

President.—How long have you had them in your possession?

R.—Not very long, and they were not consecrated by the priest. Another pack of cards, which I had owned for a long time, he consecrated for me.

President.—You must show us how you proceed in consulting your cards.

R.—Good heavens! I am too much frightened just now.

However, he took the cards, selected three from the pack, and shuffled them again with the others. He then requested the secretary of the court to cut them three times, and, sorting them in four rows of eight each (German packs contain but thirty-two cards), declared the second card in the first row to represent Catherine, and the second in the third row to stand for Lauder. "There you see that," he adds triumphantly, "they are again together." He cannot, however, find the nail of death amongst his mysterious pack.

President.—Do you believe in the existence of witches?

R.—When I was a little boy I recollect seeing a witch riding down from the clouds during a great thunderstorm. The peasants then caught and shot her on the spot. I therefore believe in witches.

President.—Why did you advise Lauder to beat and maltreat Catherine?

R.—I only told him to beat her a little.

President.—But as Lauder and his wife asked you for another remedy, why did you insist on the beating?

R.—I did not know of any other, and I firmly believed that it would effect the cure.

Lauder was sentenced to be imprisoned for life; the verdict upon the other criminal is not given in the papers.

Recitals like the foregoing enable us better to comprehend the feelings which actuated individuals in the darker and more superstitious periods of history.

